# Effects of microclimate on behavioural and life history traits of terrestrial isopods: implications for responses to climate change

**DOI:** 10.3897/zookeys.515.9399

**Published:** 2015-07-30

**Authors:** Bernice Dixie, Hollie White, Mark Hassall

**Affiliations:** 1 School of Environmental Sciences, University of East Anglia, Norwich NR4 7TJ, UK; 2 School of Biological Sciences, University of East Anglia, Norwich NR4 7TJ, UK; 3 Current address:Bernice Dixie, School of Environmental Sciences, University of East Anglia, Norwich, NR4 7TJ, UK

**Keywords:** Temperature, moisture, aggregation, growth rates, mortality rates, stimulation of micro-organisms

## Abstract

The sensitivity of terrestrial isopods to changes in both temperature and moisture make them suitable models for examining possible responses of arthropod macro-decomposers to predicted climate change. Effects of changes in both temperature and relative humidity on aggregation, growth and survivorship of species of isopods contrasting in their morphological and physiological adaptations to moisture stress have been investigated in laboratory microcosms.

All three traits were more sensitive to a reduction in relative humidity of 20–25% than they were to an increase in temperature of 5–6 °C. These results suggest that predicted changes in climate in south east England may reduce the extent to which soil animals stimulate microbial activity and hence carbon dioxide (CO_2_) emissions from soils in the future. This may help to mitigate the potential for a positive feedback between increased CO_2_ emissions from soils, and increased greenhouse effects causing an increase in soil temperatures.

## Introduction

Climate change is the greatest human induced environmental challenge ever faced by mankind (Doney et al. 2012, Middleton 2008). In South East England global climate change models predict that by 2060 air temperatures will have risen by 1.4–5.8 °C (Bale et al. 2002), summer rainfall decreased by 50% (Murphy et al. 2009) and be restricted to fewer more intense episodes with more and longer periods of drought (Rowell 2005). Such changes could lead to a reduction in relative humidity at the soil/litter interface (Edwards 2013). One potential consequence of climate change, particularly at higher latitudes, is development of a positive feedback loop, in which warmer temperatures lead to accelerated mineralisation of soil organic matter, therefore to increased emissions of carbon dioxide (CO_2_) and methane, leading to an increased greenhouse effect resulting in further increases in soil temperatures (Heimann and Reichstein 2008). Changes in microclimate may affect rates of CO_2_ emissions from soils in two ways: directly influencing microbial metabolism and indirectly by influencing the externt to which soil animals regulate microbial metabolic processes (Bardgett et al. 2008, Dias et al. 2012).

Soils contain the world’s largest terrestrial stores of carbon, releasing ten times more CO_2 _than all anthropogenic emissions combined (Schlesinger and Andrews 2000). It follows that a 1% increase in CO_2_ emitted from the soil is equivalent to a 10% increase in anthropogenic emissions (Dias et al. 2012). The majority of CO_2_ emitted from the soil is due to microbial catabolism of soil carbon pools (Lavelle 2002), but this process is influenced by soil animals as key system regulators of this process (Coleman et al. 2004). This regulation is achieved by soil animals stimulating microbial activity partly because they redistribute propagules to fresh substrate (Lavelle and Spain 2001) and partly because they can change the substrate physically, including increasing its surface area as a result of comminution (Hassall and Sutton 1977), chemically e.g. by altering pH and nitrogen (McBrayer 1973) and biologically by changing the densities and hence taxonomic composition of microbial populations and communities (Zimmer and Topp 1999).

Members of the soil macrofauna may respond to future climate conditions by both “functional responses” and “numerical reponses”, in this paper used in a broad sense of changes in their activities which may alter their “function” in the ecosystem. Specifically we use aggregation as an example of a functional response. Secondly by “numerical responses” which are those responses of life history and population parameters that might influence the density and population dynamics of individual species.

Terrestrial isopods form a dominant component of the soil macrofauna in many ecosystems (Hassall et al. 1987). They are found in a wide range of ecosystems, from xeric deserts (Warburg 1965) to temperate littoral zones (Oliver and Meechan 1993, Paoletti and Hassall 1999). Terrestrial isopods are strongly sensitive to microclimatic conditions and have developed morphological and behavioural traits, such as pleopodal lungs and aggregation, as adaptations to the terrestrial environments (Warburg 1968). The extent to which morphological traits have developed to reduce moisture loss varies between different families: for example members of the Oniscidae lack well developed pleopodal lungs in contrast to members of the Porcellionidae which do have elaborately developed pleopodal lungs with a large number of fine branched tubules, resulting in a greater surface area for absorbing oxygen with reduced loss of water vapour (Wright and Ting 2006). *Oniscus asellus* was chosen as a representative of the Oniscidae family while *Porcellio scaber* and *Porcellio dilatatus* were used, according to availability, as representatives of the Porcellionidae, both species having elaborate pleopodal lungs.

Aggregation is a behavioural adaption to avoid desiccation, where individuals group together to reduce water loss by creating a shell of higher relative humidy around the aggregate (Allee 1926, Friedlander 1964, Morris 1999, Broly et al. 2013). An isolated individual isopod can lose water three times as fast as one in an aggregation (Brockett and Hassall 2005). However, there is a cost to aggregation, as isopods do not feed while aggregating to shelter (Dias et al. 2012). This can potentially alter the extent to which they stimulate microorganism mediated mineralization of carbon based substrates (Hassall et al. 2010, Dias et al. 2012).

Microclimatic variables also strongly influence the numerical responses of isopods by affecting population characteristics such as, growth rate and survivorship (Edney 1954, Rushton and Hassall 1987). In this paper we present the effects of altering both temperature and relative humidity on species of isopods contrasting in both their morphological and aggregative responses to changes in microclimate.

We test the following hypotheses:

1. As relative humidity decreases aggregation and mortality rates will increase while growth rates will decrease.
2. As temperature increases aggregation, growth rates and mortality rates will all increase.

## Materials and methods

### Experimental design

In the experiments on responses of aggregation behaviour to different temperatures and relative humidities five treatments were used: for responses to temperature 14, 17, 19, 21 or 23 °C and for responses to relative humidity 50, 60, 70, 80 or 90%. These ranges were chosen to bracket the range of microclimate conditions that might be encountered at the litter/soil interface of soils in south east England. 58 replicate arenas were used for each set of microclimate conditions. In both experiments *Oniscus asellus* was used as a representative of the Oniscidae and *Porcellio scaber* as a representive of the Porcellionidae for investigating responses of aggregation behaviour to temperature and *Porcellio dilatatus* as a representative of the Porcellionidae for investigating responses of aggregation to relative humidity.

*Oniscus asellus* and *Porcellio dilatatus* were also used to investigate responses of relative growth rate and mortality. A 2 × 2 factorial experimental design was used with temperatures of 18.5 °C and 13.5 °C and relative humidities of 90% and 70%. Each treatment combination was replicated 10 times. Differences in temperature of 5 °C, represent close to the higher predicted increases in air temperatures during the 21^st^ century (Bale et al. 2002). As predictions of how climate change might influence relative humidity at the soil/litter interface are not yet readily available, a 20% decrease in relative humidity was chosen as being the range observed within spring and summer in the litter layer of a fixed dune grassland (Hassall 1976).

## Experimental protocols

### Responses in aggregating behaviour

For investigating responses in aggregating behaviour to temperatures, base cultures of *Oniscus asellus* and *Porcellio scaber* were kept at 23 °C in the dark in 60 × 40 × 16 cm plastic containers with sloping bases of moist plaster of Paris from 0.5–4.0 cm deep covered with 6–2 cm sand to give a level surface with a moisture gradient along the length of the box and with pieces of bark for shelter and a mixture of leaves from broad leaved trees scattered over the sand surface for food. For investigating responses of aggregation to different relative humidities *Oniscus asellus* and *Porcellio dilatatus* base cultures were kept at 23 °C in the dark in 28 × 9 × 15.5 cm plastic containers with a base of 2 cm dampened plaster of Paris covered by 3cm sand and leaf litter. A mixture of broad leaved tree species provided food and cover. The boxes were sprayed with water at approximately 2 day intervals. For both experiments a soft paintbrush and a plastic weighing boat were used to transfer 10 individuals of a single species into 90 mm diameter Petri dishes divided into eight equal sections. Petri dish lids were replaced by a piece of nylon mesh (Hassall et al. 2010).

Experimental arenas were transferred to and from the middle shelf of SANYO versatile environmental test chambers set to 90% RH for investigating responses to temperature and at 22 °C for investigating responses to relative humidity. Arenas were left for 20 minutes to allow arenas to equilibriate to experimental temperatures. They were then removed, photographed and the number of individuals in each section were counted. If an individual was on the dividing line between two segments it was recorded as in the section in which the largest percentage of its body was situated. If the individual was exactly half way over the boundary then it was recorded as being in the section in which the head end was situated (Hassall et al. 2010). After recording their dispersion in the arena, the isopods were returned to the holding container and not used again that day. The Petri dishes were wiped with 70% ethanol to remove aggregation pheromones (Devignes et al. 2011) and were not used again that day.

The variance mean: ratio for numbers in segments was used as an index of aggregation. Aggregation indices were analysed in SPSS, using a two-way ANOVA, and Tukey *post hoc* comparison of means to compare species and humidities.

### Responses of growth and mortality rates

Three males and three females of both *Oniscus asellus* and *Porcellio dilatatus*, were divided into size classes and placed into a plastic container measuring 28 × 9 × 15.5 cm (Hassall et al. 2003) lined with 2 to 3 cm of plaster of Paris covered by a 1.5 to 2 cm deep layer of damp sand (Helden and Hassall 1998). Each container had a circular 60 mm diameter wooden shelter supported by a cork above a 38 mm × 10 mm deep plaster of Paris filled Petri dish as a base and a 40 mm Petri dish lid embedded in the sand at the opposite end of the arena as a food container.

Containers were kept in SANYO versatile environmental test chambers and sprayed daily with the average mean summer rainfall of 1.65 mm (Moss and Hassall unpublished). The containers were kept in darkness for the full four weeks (Rushton and Hassall 1987). Isopods were removed from the containers and weighed individually on a Mettler B204 balance every week for four weeks. The population density was sustained throughout the four weeks by replacing any animals that died. The growth rates of the replacements were not monitored as they had not been under the experimental conditions for the same length of time as the original experimental individuals (Hassall et al. 2003).

The growth rate was calculated using relative growth rate, RGR (Helden and Hassall 1998):

RGR = [log(LW*_t1_*)- log(LW*_t0_*)]/ T

where LW*_t0 _*is the live mass (mg) at the start of the experiment, LW*_t1 _*is the live mass four weeks later at the end of the experiment and T is the number of days from the start to the end of the experiment. The number of isopods of each species that died in each container was recorded weekly. Average values for growth rates in each container were used in the analyses to avoid pseudoreplication. The data were analysed using hree-way ANOVA in SPSS following testing for normality using the Kolmogorov-Smirnov test. *Post hoc* Tukey tests were used to compare different means. Mortality rates per week were analysed using the Mann Whitney U test as the data were not normally distributed.

## Results

### Responses in aggregating behaviour

**Figure 1.**
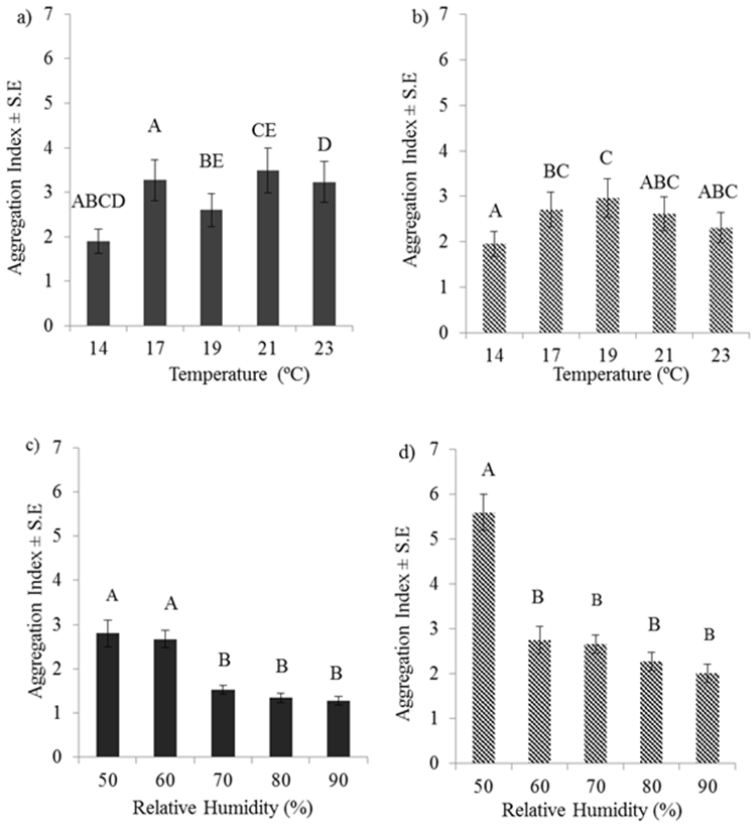
Responses in aggregation index to differences in temperatures and relative humidity: Responses to different temperatures by a) *Oniscus asellus*, (F _4, 249 _ = 12.22; P < 0.001) and b) by *Porcellio scaber* (F_4,249 _= 3.76; P < 0.001). and to different relative humidies by c) *Oniscus asellus*, (F _4, 230 _ = 25.39; P < 0.001) and d) by *Porcellio dilatatus* (F_4,171 _= 16.85; P < 0.001). Means sharing the same letter are not significantly different from each other at P < 0.05.

Differences in aggregation under different microclimate conditions for both *Oniscus asellus* and *Porcellio scaber* varied significantly with temperature. For both species the most significant increase occurred between 14 and 17 °C (Fig. 1a, b). For *Porcellio scaber* aggregation peaked at 19 °C and then decreased as the experimental animals increasingly switched from aggregating to escape behaviour: walking rapidly round the arena and climbing the walls.

In order to summarise results those for 17–23 °C were combined (average temperature 20 °C) for comparison with the significantly lower results for 14 °C. This showed that for *Oniscus asellus* aggregation was 64% higher and for *Porcellio scaber* 28% higher, following a 6 °C increase in temperature. Aggregation also changed significantly (P < 0.001) for both *Oniscus asellus* and *Porcellio dilatatus* at different relative humidities (Fig. 1c, d). For *Oniscus asellus* the significant change was between 60% and 70% relative humidity (Fig. 1c). For *Porcellio dilatatus* significant change occurred between 50% and 60% relative humidity (Fig. 1d). Summarising the results by averaging values for treatments that did not differ significantly, showed that there was an increase of 128% in the aggregation of *Oniscus asellus* and 99% increase for *Porcellio dilatatus* when relative humidity decreased by, on average, 25%.

Aggregation of *Oniscus asellus* increased by more than double the increase in aggregation shown by *Porcellio scaber* for the same change in temperature. *Oniscus asellus* also responded more to changes in relative humidity than *Porcellio scaber*. Averaging the results for the two species by combining results for the two temperatures showed that for both species combined aggregation increased by 114% for a decrease of 25% in relative humidity (average value for F = 21.1) compared with a 46% increase in aggregation for a 6 °C rise in temperature (average value for F = 8.0).

### Responses in growth and mortality rates to different microclimatic conditions

**Figure 2.**
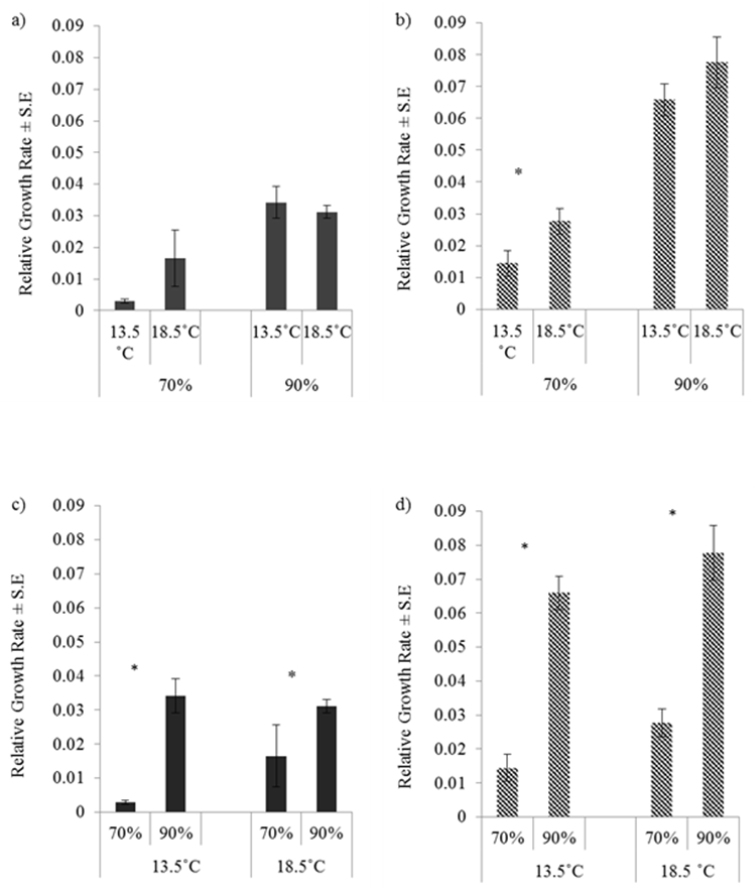
Responses of relative growth rates to temperature and relative humidity. Responses to differences in temperature by a) *Oniscus asellus*, (F_1, 36_ = 0.905, P = 0.348) and. b) by *Porcellio dilatatus*, (F_1, 36_ = 5.112, P = 0.030); to differences in relative humidity of c) *Oniscus asellus*, (F_1, 36_ = 17.125, P < 0.001) and d) *Porcellio dilatatus*, (F_1, 36_ = 84.326, P < 0.001). Asterisks denote differences signficance at P < 0.05.

Results of the 2 × 2 factorial experiment to investigate responses of growth and mortality to a 5 °C rise in temperature and a 20% reduction in relative humidity for *Oniscus asellus* and *Porcellio dilatatus* are shown in Fig. 2a–d. For only one of the four species/relative humidity combinations was there a significant increase in growth rate at the higher temperatures (at 70% RH comparing 13.5 and 18.5 °C for *Porcellio dilatatus* (Fig. 2b)) In contrast for both species at both temperatures, growth rates were significantly higher at 90% RH than at 70% RH. These results thus indicate a more consistent decrease in growth rates for a 20% decrease in relative humidity than increases in growth rates for a 5 °C increase in temperature.

**Figure 3.**
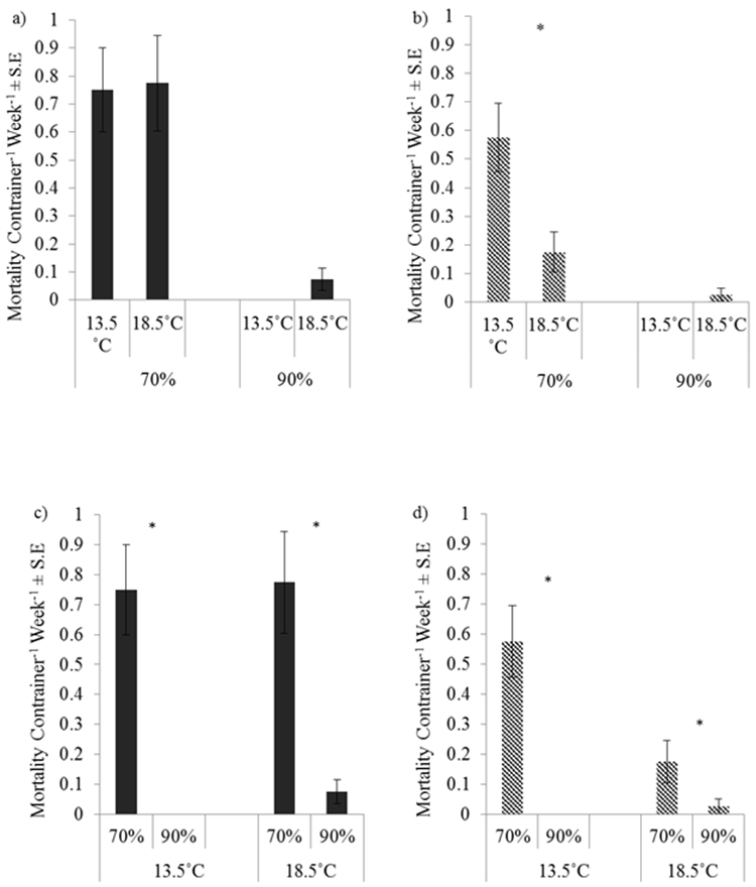
Response of mortality to temperature and relative humidity. Responses to temperature by a) *Oniscus asellus*, (U = 3097.0, P = 0.640. and b) by *Porcellio dilatatus*, (U = 2254.5, P = 0.016) and to relative humidity by c) *Oniscus asellus* (U = 1851.5, P < 0.001) and d) by *Porcellio dilatatus* (U = 2277.5 P < 0.001). Asterisks denote differences signficance at P < 0.05.

Similarly for mortality for one of the four combinations of species and relative humidity (*Porcellio dilatatus* at 70% RH) was there a significant difference between 13.5 and 18.5 °C (Fig. 3). In contrast for all four species/temperature combinations mortality was significantly higher at 70% RH than at 90% RH, again suggesting a more consistently higher response to a 20% decrease in relative humidity than for responses to a 5 °C increase in temperature.

## Discussion

In this paper we use a reductionist, experimental approach to evaluate different responses to components of climate change. Specifically we address the question: will responses to climate change by soil animals indirectly help to mitigate the direct effects of increased soil temperatures increasing microbial activity and hence microbially mediated CO_2 _emissions? These direct responses of micro-organisms to a change in global temperatures could potentially cause a positive feedback between increased atmospheric CO_2_ concentrations, increased greenhouse effects, and increases in soil temperature and microbial metabolism (Reichstein et al. 2002).

While predictions of above ground temperature and rainfall patterns are reaching a sophisticated level of both spatial and temporal resolution (IPCC 2013), predicting how these changes will affect the micro-climate of the soil/litter interface inhabited by many soil animals, is less precise due to the buffering effects of the cooling capacity of the soil and evaporation of water from within it. In order to balance obtaining significant signal to noise ratios while retaining a realistic approach to predicted changes, we have used the top end of the range of predicted increases in air temperature in the 21^st^ century for high emissions scenarios (5–6 °C) (Bale et al. 2002) and, in the absence of reliable predictions of future changes in relative humidity at the soil surface, reductions in relative humidity of 20–25% based upon observed intra-annual ranges in relative humidity in the litter layer of a fixed dune grassland (Hassall 1976). A 50% reduction of summer rainfall is predicted for south east England by 2090. The period between future summer events is also predicted to double producing longer droughts (Murphy et al. 2009). Moss (2007) found that reducing simulated rainfall in experimental mesocosms by 50% reduced soil moisture by 20%.

Isopods are used as model arthropod macro-decomposers because their physiological, behavioural and ecological responses to different microclimates are well known (Sutton 1980) and they are prominent components of the macro-decomposer fauna in many ecosystems (Davis and Sutton 1977). However there is a wide range of adaptation to the terrestrial environment between different families of the sub-order Oniscidae (Edney 1965, Warburg 1968), members of the family Oniscidea notably having much less well developed respiratory surfaces than the pleopodal lungs characteristic of members of the family Porcellionidae.

The experimental animals responded to both an increase in temperature from 14 to 20 °C and a reduction of 25% in relative humidity by increasing the degree of aggregation, as found over other temperature and relative humidity ranges (Allee 1926, Hassall et al. 2010). These components of the functional responses of isopods to predicted changes in temperature and rainfall will thus act together to reduce isopod activity, which will therefore reduce the extent to which these soil animals are likely to act as “Prince Charming” in Lavelle and Spain’s (2001) “Sleeping Beauty” analogy of how soil animals stimulate micro-organisms by transporting propagules to new substrates. Aggregative responses of isopods to predicted trends in climate change are therefore likely to partially mitigate the positive feedback cycle of increased soil temperatures and CO_2_ emissions.

Both growth and mortality rates responded consistently more to a 20% decrease in relative humidy than to a 5 °C increase in temperature. In three of the four species × humidity treatments growth rates did increase over this range of temperature, as would be expected from previous studies of isopod growth rates (Grundy and Sutton 1989) but only one of the increases was significant. In contrast for all four species × temperature comparisons a decrease of 20% in relative humidity resulted in significant decreases in relative growth rates. The same pattern was apparent for mortality rates as only one out of four changes in mortality rates in response to an increase of 5 °C was significant which was an anomalous decrease in mortality at the higher temperature. In contrast mortality responses to decreases of 20% in relative humidity were consistently significant in all four temperature × species combinations, higher mortality occurring at the lower relative humidity as found in other studies summarised by Sutton (1980). As fecundity is a function of size in terrestrial isopods (Sunderland et al. 1976) the net effect of a combination of responses for growth and mortality on the overall numerical response by isopods to climate change is therefore likely to be negative and so likely to reinforce the aggregation functional responses in reducing stimulation of soil micro-organisms. Indirect effects of climate change, via effects on stimulation of soil micro-organisms by these soil animals altering their regulation of microbial activity, would therefore reinforce the mitigation of increased microbial metabolism and hence CO_2_ emissions from soils likely to result from predicted future increases in temperature.

In conclusion the answer to the question of whether the net functional and numerical responses of these model arthropod macro-decomposers will have a negative effect on the potential positive feedback between increased soil temperatures, increased soil CO_2 _emissions increasing greenhouse warming and hence further soil warming, will vary with regional differences in future rainfall patterns. In regions predicted to experience significant decreases in levels or periodicity of rainfall, the functional role of the animals in stimulating the micro-organisms is likely to be reduced. This can be predicted to partly mitigate against the potential positive feedback that could lead to increased CO_2 _emissions from soils.
